# Some Observations on the Utility of Fumigating and Other Baths, &c.

**Published:** 1834-04-01

**Authors:** 


*Some Observations on the Utility of Fumigating and other Baths, &c.* By Jonathan Green, M.R.C.S.—London, 1834. 12mo. pp. 75.

This little work is obviously not intended for the profession, but for the public; a fact which would justify us in omitting to notice it. But the book, small though it be, may serve as a *flapper* to awaken the attention of some sleepy practitioners to the unrivalled advantages derived from warm and vapour baths in many diseases,—for example, chronic rheumatism, and a host of cutaneous affections.

We cannot help observing, that, among the disadvantages attendant on writing a medical book for the *profanum vulgus,* is to be numbered the supposed necessity of making it, like an expurgated Shakspeare or Gibbon, "fit to be read aloud in families." Thus, Mr. Green, in his list of people cured of "syphilitic complaints," tells us of one Mr. B., whose complaint was "primary ulcerations * * * occasioning destruction of parts." Next to him follows another Mr. B.. suffering from "ulceration in throat, palate, and nose, * * *." All this is truly mysterious; but perhaps we may be allowed to conjecture that these stars are like the phosphorescent light playing about decayed fish, and that they indicate the dissolution of the nose to which they belong.

However, leaving the book to the persons for whom it is intended, the system is an excellent one, and we heartily wish Mr. Green and his baths every possible success.

